# On-site testing and case management to improve hepatitis C care in drug users: a prospective, longitudinal, multicenter study in the DAA era

**DOI:** 10.1186/s12889-021-11608-9

**Published:** 2021-08-20

**Authors:** Dana Busschots, Rob Bielen, Özgür M. Koc, Leen Heyens, Eefje Dercon, Rita Verrando, Filip Janssens, Luc Van den Bergh, Peter Van Lint, Liesbeth Bruckers, Frederik Nevens, Geert Robaeys

**Affiliations:** 1 Faculty of Medicine and Life Sciences, Hasselt University, Martelarenlaan 42, 3500 Hasselt, Diepenbeek Belgium; 2 Department of Gastroenterology and Hepatology, Ziekenhuis-Oost Limburg, Genk, Belgium; 3 Medical Microbiology, School of NUTRIM, Maastricht University Medical Center, Maastricht, The Netherlands; 4 zorGGroep Zin Limburg, Hasselt, Belgium; 5 Department of Gastroenterology, Jessa Hospital, Hasselt, Belgium; 6 Department of Gastroenterology, Sint-Trudo Hospital, Sint-Truiden, Belgium; 7 Department of Gastroenterology, AZ Vesalius, Tongeren, Belgium; 8 Faculty of Science, Center for statistics, Interuniversity Institute for Biostatistics and statistical Bioinformatics, Hasselt University, Diepenbeek, Belgium; 9 Department of Gastroenterology and Hepatology, University Hospitals KU Leuven, Leuven, Belgium

**Keywords:** Hepatitis C, People who use drugs, Case management, Opioid agonist therapy, Care cascade, High-income country

## Abstract

**Background:**

Screening and treatment of hepatitis C virus (HCV) infection in people who use drugs (PWUD) remains insufficient. Reducing the burden of HCV infection in PWUD requires interventions focusing on the different steps of the HCV care cascade.

**Methods:**

We performed a prospective, multicenter study, evaluating the impact of an HCV care model on the HCV care cascade among PWUD attending an addiction care center in Belgium between 2015 and 2018. Interventions within the care model consisted of pre-test counseling, on-site HCV screening and case management services. A multiple logistic regression model was performed to identify the independent factors influencing the outcomes.

**Results:**

During the study period, 441 PWUD were registered at the addiction care center, 90% (395/441) were contacted, 88% (349/395) were screened for HCV infection. PWUD were more likely to be screened if they had ever injected drugs (*p < .001;* AOR 6.411 95% CI 3.464–11.864). In 45% (157/349), the HCV antibody (Ab) test was positive, and in 27% (94/349) HCV RNA was positive. Within the Belgian reimbursement criteria (fibrosis stage ≥ F2), 44% (41/94) were treated. Specialist evaluation at the hospital was lower for PWUD receiving decentralized opioid agonist therapy (*p = .005;* AOR 0.430 95% CI 0.005–0.380), PWUD with unstable housing in the past 6 months before inclusion (*p = .015;* AOR 0.035 95% CI 0.002–0.517) or if they were recently incarcerated (*p = .001;* AOR 0.010 95% CI 0.001–0.164).

**Conclusions:**

This HCV care model demonstrated high screening, linkage to care, and treatment initiation among PWUD in Belgium. Using the cascade of care to guide interventions is easy and necessary to monitor results. This population needs guidance, not only for screening and treatment initiation but also for the long-term follow-up since one in six had cirrhosis and could develop hepatocellular carcinoma. Further interventions are necessary to increase linkage to care and treatment initiation. Universal access to direct-acting antiviral therapy from 2019 will contribute to achieving HCV elimination in the PWUD population.

**Trial registration:**

Clinical trial registration details: www.clinicaltrials.gov (NCT03106194).

**Supplementary Information:**

The online version contains supplementary material available at 10.1186/s12889-021-11608-9.

## Background

Chronic infection with the hepatitis C virus (HCV) remains a worldwide health problem. The World Health Organization (WHO) has set a target to reduce HCV infection incidence by 90% and liver-related mortality by 65% while increasing diagnosis and treatment by 2030 with the year 2015 as baseline [1]. With the development of direct-acting antivirals (DAA), HCV treatment has become very effective [2–4].

The seroprevalence of hepatitis C virus (HCV) is relatively low (1.0%) in Belgium’s general population [5]. However, the prevalence is increased in several subpopulations and these high-risk populations can continue to transmit the virus if risk behavior persists (e.g., injecting drug) [6, 7].

Accordingly, at-risk populations such as people who use drugs (PWUD) should also be treated as they are responsible for the virus’s ongoing transmission [8].

A cascade of care is a well-known concept and pinpoints where gaps in services may exist and strategies can be developed to better support patients. However, only a few studies have evaluated the cascade of care in PWUD since the introduction of DAAs. These studies mainly focus on (active) people who inject drugs (PWID), which means that other risk subgroups such as stimulant users and former PWID are not reached [9–12]. A large study in British Colombia, Canada, showed that active PWID had the highest rates for HCV RNA testing, though the lowest for treatment initiation [13]. In addition, the treatment rates remained low in all studies, even in the DAA era.

Several barriers to HCV care may explain the lower rates of screening and treatment among drug users [14]. Studies show that both patients and caregivers lack knowledge about HCV infection, and stigmatization of HCV infection has a significant impact on admission for screening and treatment [15–17]. In a recent systematic review, interventions to enhance HCV screening, linkage to care, and treatment initiation were evaluated [18]. Two interventions were shown to improve HCV screening: (1) on-site testing with pre-test counseling and education, and (2) dried blood spot testing [18–20]. Furthermore, facilitated referral for HCV specialist evaluation and planning of specialist appointments improved linkage to care [21–23]. Integrating HCV care in addiction care centers by a multidisciplinary team with case management services can also improve treatment rates [24, 25]. However, most studies were cross-sectional, performed in the interferon-era and PWID. Therefore, more longitudinal data are necessary from the DAA era to strive towards HCV elimination in PWUD.

Before 2015, there was no systematic approach to address HCV infection among PWUD in the Center for Alcohol and Drug abuse (CAD) Limburg in Belgium. A small randomized controlled trial (RCT) was conducted in 2014 at the addiction care center by Arain et al. to assess the influence on knowledge and willingness for HCV screening and treatment among PWUD. The single information session significantly improved HCV knowledge, though it did not result in higher rates for screening and treatment. However, the small number of subjects (*n* = 52) and the fact that the study was conducted in the interferon era should be considered when interpreting this study’s results [7]. Hence, an urgent need for an easy-to-implement model was needed to offer HCV care to PWUD in an addiction care center. Therefore, we conducted this study. We offered several targeted interventions (pre-test counseling, on-site testing, facilitated referral) to positively influence the HCV cascade of care in PWUD in the addiction care center in Limburg, Belgium.

## Methods

### Study settings

PWUD are defined as people who have a history of drug use or who actively use drugs (excluding alcohol). Eligible participants were PWUD, older than 18 years, and registered at CAD Limburg between July 2015 and December 2018.

CAD Limburg is an organization providing addiction care (including opioid agonist therapy (OAT)) on several sites throughout the province of Limburg, Belgium. OAT is provided on-site (central provision) or is prescribed by the addiction care physician. The clients who only receive a prescription for OAT can receive their treatment at a local pharmacy (decentralized provision). Before 2015, there was no systematic approach to address HCV infection among PWUD at this facility for addiction care.

Between 2015 and 2017, DAA regimens were only reimbursed if fibrosis was staged ≥ F3 in Belgium [26]. As of January 2017, the reimbursement criteria have been adjusted to ≥ F2. Unlimited access has only been possible since 2019 [27, 28]. Besides, DAA treatment can only be prescribed and initiated by a hepatologist and is available only in a hospital pharmacy.

### Care model

This is a longitudinal, multicenter study with a stepwise implementation of services to improve HCV care in PWUD.

An overview of the services provided and the changing treatment landscape throughout the study period is provided in (see Additional file 1). In the first phase (July 2015 to November 2016), one medical PhD student functioning as case manager offered on-site information on HCV infection and immediate screening by venipuncture to attending clients. Results of screening were explained on-site, and PWUD with positive HCV RNA were referred to one of the two hospitals prescribing DAA treatment at that time in Limburg.

In the second phase (December 2016 to December 2018), one nurse functioning as case manager provided these services. On a weekly basis the case manager was at the locations with central provision and at the centers with decentralized provision by appointment. The HCV case manager actively contacted clients, informed them about HCV infection and offered to test them for HCV. If a chronic HCV infection was diagnosed after blood sampling, a facilitated referral was suggested to guide the PWUD to the hospital. The case manager had regular contact with the clients and sent reminders when they were expected on an appointment.

From 2017 onwards, PWUD could be referred to all hospitals in Limburg. HCV finger-prick tests (OraQuick® HCV Ab) were provided to replace screening by venipuncture in 2018, though HCV RNA was still assessed by venipuncture [29, 30]. A FibroScan® was performed to assess liver damage in HCV RNA-positive patients who reached the hospital for a specialist evaluation (cutoffs for HCV/HIV: F0-F1 = < 7.2 kPa, F2 = 7.2–9.5 kPa, F3 = 9.5–11.5 kPa, F4= > 11.5 kPa) [31].

The study was approved by the Ethical Committees of Ziekenhuis Oost-Limburg, Genk, and Hasselt University (16/014 U). An amendment was approved to include all hospitals’ study sites in 2017 and perform the services by a nurse as a case manager. The study protocol was registered at clinicaltrials.gov (NCT03106194). The study was conducted following the provisions of the Declaration of Helsinki and its amendments. Good clinical practice (GCP) guidelines were followed throughout the study [32].

### Data collection and outcomes

Upon informed consent, a yearly face-to-face questionnaire was completed and subsequently stored using an online e-CRF software tool (Castor EDC). Screening for HCV infection was executed and analysis for HCV Ab was performed using a third-generation ELISA assay (Abbott HCV 3.0 R, Abbott Diagnostic, Chicago, IL, USA). If HCV Ab positivity was confirmed, reflex HCV RNA testing was done using a quantitative real-time polymerase chain reaction (qRT-PCR).

There was no difference in the recruitment method between people at centralized sites compared to those at decentralized sites. An annual face-to-face questionnaire assessed continued risk behavior since the last study visit. For the analyses in this study, only the data from the questionnaire at the time of inclusion was used. In case of continued risk behavior, yearly HCV screening was offered. Risk behavior was only defined as active injection drug use since the last visit. The treating hepatologists filled out treatment characteristics and outcomes. The loss to follow-up (LTFU) was defined as loss of contact with the PWUD despite at least three attempts. All data were encoded and stored according to GCP practices.

### Endpoints of the study

The primary objective of this study was to construct an HCV cascade of care for PWUD attending an addiction care center by implementing pre-test counseling, on-site testing and facilitated referral. By building a care cascade, the strengths and weaknesses of HCV care in Belgian PWUD can be exposed. The secondary objective of this study was to identify risk factors influencing this cascade of care.

### Statistical analysis

Statistical analysis was performed using SPSS version 24. The cascade steps were determined: PWUD contacted for screening, screened, HCV antibody (Ab) positive, HCV RNA positive, specialist evaluation, started treatment and cured. To study baseline characteristics influencing rates of screening, diagnosis of chronic HCV infection, assessment and treatment initiation, univariate analyses were performed using the Chi-square test for categorical data. Continuous data were tested for normality and homogeneity of variances by Shapiro-Wilk and Levene values of *p > .050*. Since age at inclusion, age at the start of drug use, and duration of intravenous drug use were not normally distributed, the Mann-Whitney U test was used to compare continuous variables. A multiple logistic regression model with a backward selection procedure was performed to identify the independent factors influencing the outcomes. Risk factors with *p < .150* in the univariate analyses were included in the model for each outcome parameter. Exploratory, we assessed differences in the cascade of care between 2015 and 2016, 2017, and 2018. A Chi-squared test or Fisher’s exact test was used on the proportions of each step in the cascade.

To demonstrate the efficacy of this screening method, with an α (significance) of 0.05, statistical power of 0.8, and *n* = 300, we will need to improve screening from 9% (26/300 patients screened before 2015) to 16% [33–35].

This manuscript only shows the results of the analyses for uptake of screening and specialist evaluation. All other variables studied in this research can be found in (see Additional file 2) and (see Additional file 3).

## Results

### Cascade of care for hepatitis C for PWUD

**Fig. 1:**
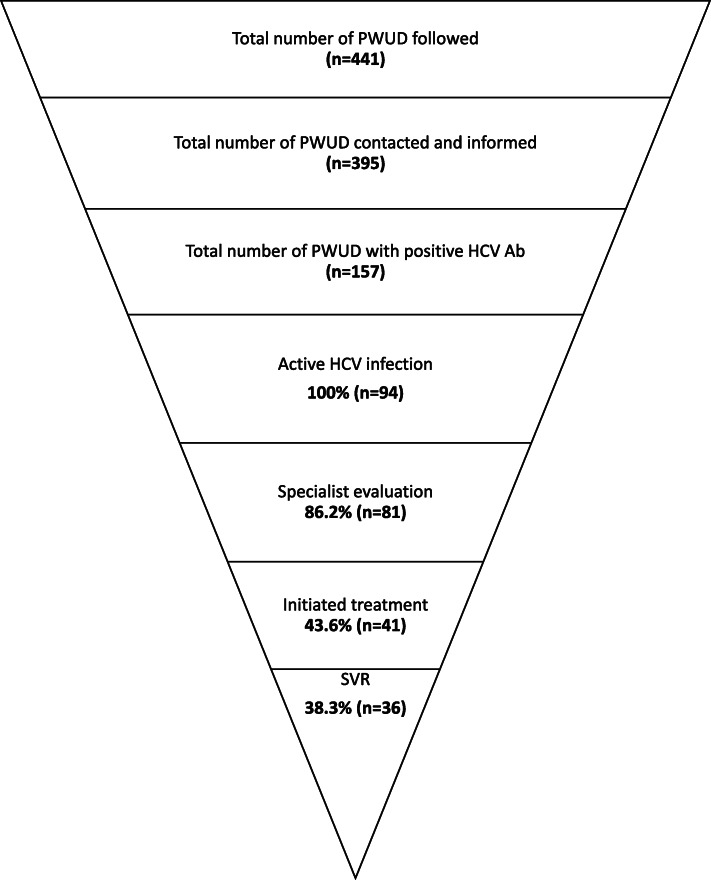
The cascade of care for hepatitis C infection for people who use drugs in Limburg. Specialist evaluation of liver disease using FibroScan® was provided to 81 patients. Within the Belgian reimbursement criteria (fibrosis stage ≥ F2), 41 patients were treated during the study period. Abbreviations: HCV hepatitis C virus; Ab = antibodies; PWUD = people who use drugs; SVR = sustained virologic response

Between 2015 and 2018, 89.6% (395/441) of the PWUD were contacted and informed regarding HCV infection. Of those, 88.4% (349/395) were screened for HCV, 45.0% (157/349) were HCV Ab positive, and 26.9% (94/349) had a positive HCV RNA test. Figure 1 illustrates the outcome for linkage to care, treatment, and outcome for HCV RNA positive patients between 2015 and 2018. Out of 21 individuals with information on continued risk behavior and HCV screening result, there was one (4.8%) known reinfection.

A higher number of PWUD were contacted for screening in 2018 (*p = .039*) compared to the other years. No significant differences were observed with the specialist evaluation (*p = .365*) or initiation of antiviral treatment (*p = .388*). During the study period, a significant decrease in HCV RNA positivity was observed (59.2% in 2015–2016, 52.8% in 2017, and 40.2% in 2018, *p = .013*).

#### Eligibility for treatment and follow-up

FibroScan® scores were available for 82.7% (67/81) PWUD who visited the hospital for specialist evaluation. METAVIR scores of F0-F1, F2, F3, and F4 were found in respectively 27 (40.3%), 14 (20.9%), 15 (22.4%), and 11 (16.4%) persons. Of those who reached the hospital for specialist evaluation, 40.3% (27/67) were not eligible for treatment within the study period. Cirrhosis (F4) was present in 16.4% (11/67).

### Patient population

In total, 441 PWUD were registered at CAD Limburg between 2015 and 2018. The total number of PWUD fluctuated over time: 312 PWUD were in follow-up in 2015–2016, 324 PWUD in 2017, and 346 PWUD in 2018. Baseline characteristics are provided in Table 1 and (see Additional file 3). Almost all PWUD had access to the Belgian health care system due to the mandatory healthcare insurance and thus had access to HCV care within the Belgian reimbursement criteria. In total, 78.0% (344/441) of the clients admitted to having injected drugs in the past, 32.8% (113/344) injected in the past 6 months. The majority of PWUD received OAT (86.6%, 382/441). PWUD receiving decentralized OAT were less likely to have injected drugs (73% vs. 87%; *p = .001*), were more often employed (31% vs. 14%; *p = .005*) and had more often stable housing (80% vs. 64%; *p = .009*) compared to individuals receiving centralized OAT.

**Table 1:** Baseline characteristics and univariate analyses for screening of people who use drugs at the Center for Alcohol and Drug abuse in Limburg

		Univariate analyses	
		Screened for HCV ***n*** = 349	
Characteristic (***n*** = 441)	N (%)	n (n/N%)	***p***-value
**Age** (mean ± SD)	42 ± 9 (19–62)	43 ± 9	.377
**Gender**			**.089**
Male	356 (80.7)	276 (77.5)	
Female	85 (19.3)	73 (85.9)	
**Source of income**			**.008**
Employment	89 (20.2)	60 (67.4)	
Welfare check	312 (70.7)	259(83.0)	
Pension	1 (0.2)	1(100)	
None	30 (6.8)	21 (70.0)	
*Missing*	*9 (2.0)*		
**Housing last 6 months**			0.859
At home (owned/rented)	311 (70.5)	243 (78.1)	
At the house of family/friends	79 (17.9)	65 (82.3)	
Prison	9 (2.0)	8 (88.9)	
Mental health/drug abuse institution	17 (3.9)	13 (76.5)	
Streets/squatted building	17 (3.9)	13 (76.5)	
*Missing*	*8 (1.8)*		
**Contact location for recruitment**			**<.001**
Centralized OAT (CAD Limburg)	211 (47.8)	187 (88.6)	
Decentralized OAT (pharmacy)	187(42.4)	121 (64.7)	
NSP	16 (3.6)	15 (93.8)	
Former PWUD	16 (3.6)	15 (93.8)	
Active user, no therapy	11 (2.5)	11 (100)	
**Ever been incarcerated**			**0.018**
Yes	234 (53.1)	198 (84.6)	
No	147 (33.3)	110 (74.8)	
*Missing*	*60 (13.6)*		
**Incarcerated last 6 months**			0.828
Yes	22 (9.4)	19 (86.4)	
No	208 (90.6)	176 (84.6)	
*Missing*	*4 (1.7)*		
**Alcohol abuse**			**0.019**
Active	144 (32.7)	124 (86.1)	
Former	25 (5.7)	23 (92.0)	
No	153 (34.7)	146 (95.4)	
*Missing*	*119 (27.0)*		
**Ever IDU**			**<.001**
Yes	344 (78.0)	302 (87.7)	
No	75 (17.0)	40 (53.3)	
*Missing*	*22 (5.0)*		
**IDU during the last 6 months**			**<.001**
Yes	113 (32.8)	105 (92.9)	
No	328 (67.2)	244 (74.4)	
**Connected to NSP**			**.007**
Yes	62 (15.1)	59 (95.2)	
No	317 (77.1)	258 (81.4)	
*Missing*	*32 (7.8)*		

*Abbreviations*: *SD* standard deviation, *OAT* opioid agonist therapy, *CAD* center for alcohol and drug abuse, *NSP* needle syringe program, *IDU* intravenous drug use, *NSP* needle syringe program

### Factors influencing the cascade of care

The adjusted odds ratio (AOR) for screening uptake in PWUD were highest among those who ever injected drugs (*p < .001*; AOR 6.411 95% CI 3.464–11.864). PWUD receiving decentralized OAT were less likely to be screened (*p < .001;* AOR 0.313 95% CI 0.167–0.588). Specialist evaluation at the hospital was lower for PWUD receiving decentralized OAT (*p = .005;* AOR 0.430 95% CI 0.005–0.380), with unstable housing in the past 6 months before inclusion (*p = .015;* AOR 0.035 95% CI 0.002–0.517) or if they were recently incarcerated (*p = .001;* AOR 0.010 95% CI 0.001–0.164). No independent factors were associated with the diagnosis of chronic HCV infection or the start of treatment (see Additional file 4).

### Loss to follow-up

In total, 83 of the 395 reached PWUD (21.0%) got LTFU. Notably, 19 PWUD who were LTFU had a positive HCV RNA test. Of these PWUD, 13 reached the hospital once for specialist evaluation during the study period and were traceless after. Two were eligible for treatment, while the others were not. For nine of these 13 patients, the reason for LTFU was known: one patient died, two patients were long-term convicted, one was in long-term rehabilitation, two moved to another city/country, two relapsed in active drug use without OAT, one was no longer connected to the OAT center.

## Discussion

More data are needed within the DAA era to identify strategies to enhance HCV testing, linkage to care, and treatment to reach the WHO’s goals [18]. This study presents an HCV care model, led by one case manager at an OAT center with different sites in Limburg, Belgium. The case manager contacted 90% of the PWUD attending the addiction care center and could screen 88% for HCV infection between 2015 and 2018. Thus, we exceed the 16% target, which shows that this care model’s implementation is very effective for HCV screening in PWUD in addiction care centers. Specific and novel to our multicenter model is that the case manager moved between the different sites to include PWUD actively and do an annual follow-up. Most importantly, due to the work of only one person, this care model has a tremendously positive and potentially enduring impact on HCV care among substance users across an entire province in Belgium.

Services such as information and education on HCV infection were integrated at the OAT center. Targeted on-site HCV screening was carried out at all the different locations of the OAT center. Previous studies to enhance uptake for screening by on-site testing with pre-test counseling varied widely, with testing rates from 18 to 86% in the interferon era [18, 36]. Nevertheless, we reported results in the DAA era, and our yearly screening rates varied from 76 to 86%, with a combined screening uptake of 89% over the 3 years. Moreover, as we compare our new results with the few data we have from before implementing this care model, the uptake of screening has increased enormously (from 9.5 to 89%). Our results point to a large, positive impact and the need to continue this care model.

In Belgium, DAA treatment can only be prescribed by a hepatologist. Therefore, specialist evaluation needed to be performed in the hospital. To enhance linkage to care, case management services were provided to facilitate referral and appointments, and PWUD were also accompanied to the hospital if requested. Internationally, studies showed increased linkage to care ranging from 51 to 82% in studies with on-site screening, education, and/or case management services [22, 23, 25, 36]. In our setting, specialist evaluation ranged from 69 to 77% yearly, with a combined linkage to care of 86% over 3 years.

Treatment initiation is the most difficult to compare with international findings. This differs the most due to novel, effective and safe treatments compared to the interferon era. Furthermore, the presence of (changing) reimbursement criteria makes a comparison between countries difficult [37, 38]. Previous studies in the interferon era demonstrated the effectiveness of interventions such as nurse-facilitated referral to the hospital and integrating HCV care on-site with non-invasive liver disease assessment and motivational education. Treatment rates ranged from 7 to 38% in these studies [21, 24, 39]. Nevertheless, we report results in the DAA era, and yearly treatment initiation ranged from 16 to 33%, with a combined treatment rate of 44%. The most important reason for refusal of therapy was located at the level of the health care system. First, 14% of all HCV RNA positives did not reach the hospital for specialist evaluation (no linkage to care). Additionally, 40% of those who reached the hospital for specialist evaluation did not reach the reimbursement criteria of a fibrosis stage ≥ F3 (2015) or ≥ F2 (2017). Of those eligible for treatment, five PWUD were refused therapy due to non-adherence. The decision to refuse therapy was always made with a multidisciplinary team (hepatologist, case manager and CAD staff). Three PWUD were incarcerated before therapy could be initiated. In Belgium, the Department of Justice regulates the prison system and not the Department of Health. Therefore, we could not initiate treatment for these patients under the supervision of the case manager. Currently, DAA treatment can only be prescribed and initiated by a hepatologist and is available only in a hospital pharmacy. If in the future, DAA therapy could be prescribed by other healthcare professionals and be available in local pharmacies, treatment access would improve, and patients would be able to receive their medication more conveniently.

PWUD on centralized OAT provision were more likely to be screened and linked to care than PWUD receiving decentralized OAT. Our intervention was implemented at the OAT center, so patients who visited often would more easily benefit from the case manager’s support. Also, PWUD receiving decentralized OAT were less likely to have injected drugs, were more often employed, and had more often stable housing. Housing instability is associated with high-risk injection behavior and impacts social networks [17, 40, 41]. Outreach will be implemented during the next years to improve screening and linkage to care for PWUD in decentralized OAT.

There were limitations to this study. Our study population consisted of PWUD, who were in contact with the OAT center. Therefore, they could already be more engaged in care and might not represent the whole PWUD population. Furthermore, our study was not an RCT due to the goal of providing HCV care to the whole population. Except for the small historical group studied by Arain et al. in 2014, there was no comparison possible, and the impact of the various steps of our care model could not be assessed well. As there was little to no data available from the period before our study intervention, a long-term comparison was difficult. Finally, we did not collect data on coinfections, which could potentially influence screening and treatment initiation.

## Conclusions

Using the cascade of care to guide interventions is easy and necessary to monitor results. Since 2019, this project has been recognized and financially supported by the Flemish government. This population needs guidance, not only for screening and treatment but also for the long-term follow-up since one in six had cirrhosis and could develop HCC. Further interventions are necessary to increase linkage to care and treatment initiation. Universal access to DAA therapy from 2019 onwards will contribute significantly to reaching HCV elimination in our cohort.

## Supplementary Information

**Additional file 1.** Changing the landscape of hepatitis C virus infection treatment and services provided to people who use drugs.

**Additional file 2.** Univariate analysis by Chi^2^ test or Mann-Whitney U test for characteristics of screened, RNA positive, specialist evaluation and treated people who use drugs.

**Additional file 3.** A multivariate regression model with backward conditional removal of variables.

**Additional file 4.** Baseline characteristics of people who use drugs at the Centre for Alcohol and Drug abuse in Limburg.

## Data Availability

All data generated or analyzed during this study are included in this published article and its additional files.

## Abbreviations

HCV: Hepatitis C virus
WHO: World Health Organization
DAA: Direct-acting antivirals
PWUD: People who use drugs
PWID: People who inject drugs
CAD: Center for Alcohol and Drug abuse
RCT: Randomized controlled trial
OAT: Opioid agonist therapy
GCP: Good clinical practice
LTFU: Loss to follow-up
Ab: Antibody
AOR: Adjusted odds ratio
