# Supplementary material for: On-site testing and case management to improve hepatitis C care in drug users: a prospective, longitudinal, multicenter study in the DAA era
Source: BMC Public Health. 2021 Aug 20;21:1574. doi: 10.1186/s12889-021-11608-9 (PMC8379886; doi:10.1186/s12889-021-11608-9)
Supplement: Supplementary file 1 — Additional file 1. Changing the landscape of hepatitis C virus infection treatment and services provided to people who use drugs. [file 12889_2021_11608_MOESM1_ESM.docx]

Additional File 1

**A1.** Changing landscape of hepatitis C virus infection treatment and services provided to people who use drugs

|  | **2015-2016** | **2017** | **2018** |
| --- | --- | --- | --- |
| Case manager | Medical PhD student | **nurse** | nurse |
| **Services** | Personal on-site information on HCV infection | Personal on-site information on HCV infection | Personal on-site information on HCV infection |
|  | On-site screening | On-site screening | On-site screening |
|  | Venipuncture | Venipuncture | Venipuncture |
|  |  |  | **HCV Ab OraQuick® finger prick test** |
|  | Targeted screening using database | Targeted screening using database | Targeted screening using database |
|  | Contact PWUD at time of methadone provision/prescription | Contact PWUD at time of methadone provision/prescription | Contact PWUD at time of methadone provision/prescription |
|  |  | **Contact PWUD registered at CAD by telephone to make screening appointments** | Contact PWUD registered at CAD by telephone to make screening appointments |
|  | On-site discussion of screening results | On-site discussion of screening results | On-site discussion of screening results |
|  | Referral to two hospitals | **Referral to all hospitals of Limburg** | Referral to all hospitals of Limburg |
|  |  | **Accompany PWUD to appointments with hepatologists** | Accompany PWUD to appointments with hepatologists |
|  | Inform and follow-up for reinfection | Inform and follow-up for reinfection | Inform and follow-up for reinfection |
| **Reimbursement criteria^36,37^** | DAA treatment available starting from fibrosis stage ≥ F3 ^42^ | **DAA treatment available starting from fibrosis stage ≥ F2 or if at risk for accelerated fibrosis progression** ^43^ | DAA treatment available starting from fibrosis stage ≥ F2 or if at risk for accelerated fibrosis progression ^43^ |
| **Available DAA treatments in Belgium and in order of preferences according to EASL guidelines^44-46^** | 1. simeprevir + sofosbuvir | 1. daclatasvir + sofosbuvir | 1. velpatasvir + sofosbuvir ± voxilaprevir |
|  | 2. daclatasvir + sofosbuvir | 2. ledipasvir + sofosbuvir | 2. grazoprevir + elbasvir |
|  | 3. Ledipasvir + sofosbuvir | 3. velpatasvir + sofosbuvir | 3. pibrentasvir + glecaprevir |
|  | 4. ombitasvir + paritaprevir + ritonavir ± dasabuvir | 4. grazoprevir + elbasvir | 4. ledipasvir + sofosbuvir |
|  |  | 5. ombitasvir + paritaprevir + ritonavir ± dasabuvir | 5. ombitasvir + paritaprevir + ritonavir ± dasabuvir |

Abbreviations: HCV: hepatitis C virus, Ab: antibody, DAA: direct-acting antiviral, PWUD: people who use drugs, CAD: Center for Alcohol and Drug abuse, EASL: European Association for the Study of the Liver
