# Supplementary material for: On-site testing and case management to improve hepatitis C care in drug users: a prospective, longitudinal, multicenter study in the DAA era
Source: BMC Public Health. 2021 Aug 20;21:1574. doi: 10.1186/s12889-021-11608-9 (PMC8379886; doi:10.1186/s12889-021-11608-9)
Supplement: Supplementary file 2 — Additional file 2. Univariate analysis by Chi2 test or Mann-Whitney U test for characteristics of screened, RNA positive, specialist evaluation and treated people who use drugs. [file 12889_2021_11608_MOESM2_ESM.docx]

Additional File 2

**A2.** Univariate analysis by Chi² test or Mann-Whitney U test for characteristics of screened, RNA positive, specialist evaluation and treated people who use drugs.

| **Characteristic** | **Screened**  **p-value; N (%);** | **RNA positive**  **p-value; N (%);** | **Specialist evaluation**  **p-value; N (%);** | **Treated**  **p-value; N (%);** |
| --- | --- | --- | --- | --- |
| **Age** (years) mean ± SD | .377 | .358 | .984 | **.006** |
| Yes  No | 43 ± 9  42 ± 8 | 46 ± 8  47 ± 7 | 46 ± 8  46 ± 7 | 48 ± 7  44 ± 8 |
| **Gender** | **.089** | .705 | .982 | .236 |
| Male  Female | 276/356 (77.5)  73/85 (85.9) | 75/119 (63.0)  19/32 (59.4) | 65/77 (84.4)  16/19 (84.2) | 35/75 (46.7)  6/19 (31.6) |
| **Contact location for recruitment** | **<.001** | .732 | **.023** | .429 |
| Centralized OAT (CAD Limburg)  Decentralized OAT (pharmacy)  NSP  Former PWUD  Active user, no therapy | 187/211 (88.6)  121/187 (64.7)  15/16 (93.8)  15/16 (93.8)  11/11 (100) | 49/78 (62.8)  29/51 (56.9)  5/6 (83.3)  9/13 (69.2)  2/3 (66.7) | 45/50 (90.0)  20/30 (66.7)  5/5 (100)  9/9 (100)  2/2 (100) | 19/49 (38.8)  14/29 (48.3)  1/5 (20.0)  6/9 (66.7)  1/2 (50.0) |
| **Source of income last six months** | **.008** | .590 | .333 | .468 |
| Employment  Welfare check  Pension  None | 60/89 (67.4)  259/312 (83.0)  1/1 (100)  21/30 (70.0) | 8/15 (53.3)  79/127 (62.2)  -  6/8 (75.0) | 6/8 (75.0)  70/81 (86.4)  -  4/6 (66.7) | 5/8 (62.5)  33/79 (41.8)  -  2/6 (33.3) |
| **Housing last six months** | .859 | **.078** | **.007** | .287 |
| At home (owned/rented)  At house of family/friends  Prison  Mental health/drug abuse institution  Streets/squatted building | 243/311 (78.1)  65/79 (82.3)  8/9 (88.9)  13/17 (76.5)  13/17 (76.5) | 59/104 (56.7)  15/24 (62.5)  6/7 (85.7)  8/8 (100)  6/8 (75.0) | 55/61 (90.2)  12/15 (80.0)  3/6 (50.0)  8/8 (100)  3/6 (50.0) | 30/59 (50.8)  6/15 (40.0)  1/6 (16.7)  3/8 (37.5)  1/6 (16.7) |
| **Level of education** | .452 | .984 | .795 | .500 |
| Primary school (7-12 years)  Partly high school (<16 years)  Completed high school (18 years)  Higher education (>18 years) | 17/18 (94.4)  107/132 (81.1)  179/226 (79.2)  6/7 (85.7) | 7/11 (63.6)  34/54 (63.0)  42/66 (63.6)  1/2 (50.0) | 6/7 (85.7)  31/34 (91.2)  37/44 (84.1)  1/1 (100) | 4/7 (57.1)  17/34 (50.0)  16/26 (38.1)  0/1 (0.0) |
| **Have you ever been incarcerated?** | **.018** | **.008** | .228 | .582 |
| Yes  No | 198/234 (84.6)  110/147 (74.8) | 73/107 (68.2)  11/27 (40.7) | 65/75 (86.7)  8/11 (72.7) | 33/73 (45.2)  4/11 (36.4) |
| **Incarcerated last six months?** | .828 | **.085** | **.004** | **.077** |
| Yes  No | 19/22 (86.4)  176/208 (84.6) | 10/11 (90.9)  62/95 (65.3) | 6/10 (60.0)  59/64 (92.2) | 2/10 (20.0)  31/62 (50.0) |
| **Current OAT?** | .185 | .977 | .444 | .687 |
| Methadone  Suboxone  None | 286/368 (77.7)  13/14 (92.9)  47/55 (85.5) | 77/123 (62.6)  4/6 (66.7)  13/21 (61.9) | 65/79 (82.3)  4/4 (100)  12/13 (92.3) | 32/77 (41.6)  2/4 (50.0)  7/13 (53.8) |
| **Ever injected drugs?** | **<.001** | .816 | .614 | .193 |
| Yes  No | 302/344 (87.7)  40/75 (53.3) | 88/142 (62.0)  4/6 (66.7) | 76/90 (84.4)  3/4 (75.0) | 37/88 (42.0)  3/4 (75.0) |
| **Injected drugs during last six months?** | **<.001** | .765 | **.052** | .941 |
| Yes  No | 105/113 (92.9)  244/328 (74.4) | 34/56 (60.7)  60/95 (63.2) | 32/34 (94.1)  49/62 (79.0) | 15/19 (44.1)  26/60 (43.3) |
| **Do you use NSP?** | **.007** | **.144** | .312 | **.061** |
| Yes  No | 59/62 (95.2)  258/317 (81.4) | 23/32 (71.9)  58/101 (57.4) | 22/23 (95.7)  53/60 (88.3) | 7/23 (30.4)  31/58 (53.4) |
| **Alcohol abuse?** | **.019** | .704 | .296 | .707 |
| Active  Former  No | 124/144 (86.1)  23/25 (92.0)  146/153 (95.4) | 40/66 (60.6)  5/9 (55.6)  38/57 (66.7) | 37/41 (90.2)  5/6 (83.2)  37/38 (92.9) | 18/40 (45.0)  3/5 (60.0)  20/38 (49.4) |
| **Duration IDU** (years); mean ± SD | .219 | .359 | .774 | .756 |
| Yes  No | 12 ± 10  16 ± 12 | 17 ± 10  15 ± 11 | 17 ± 10  16 ± 14 | 17 ± 10  16 ± 11 |

Univariate analysis per category of the case manager's work: screened, diagnosed with chronic HCV infection, assessed for treatment and treated. All variables with a p-value <0.15 (**in bold**) were included in a multivariate regression model per category (Additional File 4). In the case of variables with collinearity (such as ever incarcerated and incarcerated last six months), only one variable was implemented in the model per analysis.

Abbreviations: SD: standard deviation; OAT: opiate agonist therapy; NSP: needle syringe program; PWUD: people who use drugs; IDU: intravenous drug use.
