# Supplementary material for: On-site testing and case management to improve hepatitis C care in drug users: a prospective, longitudinal, multicenter study in the DAA era
Source: BMC Public Health. 2021 Aug 20;21:1574. doi: 10.1186/s12889-021-11608-9 (PMC8379886; doi:10.1186/s12889-021-11608-9)
Supplement: Supplementary file 3 — Additional file 3. A multivariate regression model with backward conditional removal of variables. [file 12889_2021_11608_MOESM3_ESM.docx]

Additional File 3

**A3.** A multivariate regression model with backward conditional removal of variables.

| **Characteristic** | **Screened** | | **Specialist evaluation** | |
| --- | --- | --- | --- | --- |
|  | **p-value** | **AOR (95% CI)** | **p-value** | **AOR (95% CI)** |
| **Contact location**  Centralized OAT (CAD Limburg)  Decentralized OAT (pharmacy)  NSP  Former PWUD  Active user, no therapy | .008  **<.001**  .550  .887  .999 | -  0.313 (0.167-0.588)  0.519 (0.060-4.459)  1.168 (0.138-9.895)  - | .092  **.005**;  .999  .999  .999 | -  0.43 (0.005-0.380)  -  -  - |
| **Ever injected drugs (**Yes) | **<.001** | 6.411 (3.464-11.864) | - | - |
| **Housing last six months**  At home (owned/rented)  At family/friends  Prison  Mental health/drug abuse institution  Streets/squatted building | -  -  -  -  - | -  -  -  -  - | .025  .211  **.001**  .999  **.015** | **-**  0.314 (0.51-1.928)  0.010 (0.001-0.164)  -  0.035 (0.002-0.517) |

Abbreviations: CI: confidence interval; AOR: adjusted odds ratio; OAT: opiate agonist therapy, CAD: Center for Alcohol and Drugs; NSP: needle syringe program; PWUD: people who use drugs.
