# Supplementary material for: On-site testing and case management to improve hepatitis C care in drug users: a prospective, longitudinal, multicenter study in the DAA era
Source: BMC Public Health. 2021 Aug 20;21:1574. doi: 10.1186/s12889-021-11608-9 (PMC8379886; doi:10.1186/s12889-021-11608-9)
Supplement: Supplementary file 4 — Additional file 4. Baseline characteristics of people who use drugs at the Centre for Alcohol and Drug abuse in Limburg. [file 12889_2021_11608_MOESM4_ESM.docx]

Additional File 4

**A4.** Baseline characteristics of people who use drugs at the Centre for Alcohol and Drug abuse in Limburg.

| **Characteristic (n=441)** | **N (%)** |
| --- | --- |
| **Age** (years) mean ± SD (range) | 42 ± 9 (19-62) |
| **Gender** (male) | 356 (80.7) |
| **Source of income last six months** |  |
| Employment  Welfare check  Pension  None  *Missing* | 89 (20.2)  312 (70.7)  1 (0.2)  30 (6.8)  *9 (2.0)* |
| **Housing last six months** |  |
| At home (owned/rented)  At family/friends  Prison  Mental health/drug abuse institution  Streets/squatted building  *Missing* | 311 (70.5)  79 (17.9)  9 (2.0)  17 (3.9)  17 (3.9)  *8 (1.8)* |
| **Level of education** |  |
| Primary school (7-12 years)  Partly completed high school (<16 years)  Completed high school (18 years)  Higher education (>18 years)  *Missing* | 18 (4.1)  132 (29.9)  226 (51.2)  7 (1.6)  *58 (13.1)* |
| **Health insurance** (yes) | 417 (94.6) |
| **Ever been incarcerated**  Incarcerated last six months | 234 (53.1)  22/234 (9.4) |
| **Current OAT** |  |
| Methadone  Suboxone  None  Missing | 368 (83.4)  14 (3.2)  55 (12.5)  4 (0.9) |
| **Contact location for recruitment** |  |
| Centralized OAT (CAD Limburg)  Decentralized OAT (pharmacy)  NSP  Former PWUD  Active user, no therapy | 211 (47.8)  187(42.4)  16 (3.6)  16 (3.6)  11 (2.5) |
| **Drug use** |  |
| Age first drug use (years) mean ± SD (range)  Ever injected drugs  IDU during last six months  Connected to NSP  Duration IDU (years); mean ± SD (range) | 19 ± 5 (10-44)  344 (78.0)  113/344 (32.8)  62 (15.1)  12 ± 10 (0-39) |
| **Alcohol abuse** |  |
| Active  Former  No  Missing | 144 (32.7)  25 (5.7)  153 (34.7)  119 (27.0) |

Abbreviations: SD: standard deviation; OAT: opiate agonist therapy; CAD: Center for Alcohol and Drug abuse; IDU: intravenous drug use; NSP: needle syringe program; PWUD: people who use drugs
